# Evaluation of the pH and Antibacterial Efficacy of Mineral Trioxide Aggregate With and Without the Incorporation of Titanium Tetrafluoride

**DOI:** 10.7759/cureus.64385

**Published:** 2024-07-12

**Authors:** Poojitha Suganthakumar, Tripuravaram Vinay Kumar Reddy, Vijay Venkatesh, Kingston Chellapandian, Mahalakshmi Krishnan

**Affiliations:** 1 Conservative Dentistry and Endodontics, SRM Kattankulathur Dental College and Hospital, SRM Institute of Science and Technology (SRMIST), Chengalpattu, IND; 2 Microbiology, Sree Balaji Dental College and Hospital, Chennai, IND

**Keywords:** mineral trioxide aggregate (mta), ph, antibacterial efficiency, enterococcus faecalis (e. faecalis), titanium tetrafluoride

## Abstract

Introduction

Microorganisms play an important role in causing inflammation in the pulp and periapical regions. Even after undergoing chemo-mechanical procedures during root canal treatment, bacteria may persist within dentinal tubules, posing a risk of disease recurrence. Mineral trioxide aggregate (MTA), introduced as a dental material, has been investigated as a potential antibacterial agent since its early use. Calcium and phosphorus are the primary ions in MTA, and their antibacterial characteristics are attributed to the release of calcium hydroxide through surface hydrolysis of calcium silicate components. Previous studies have shown that MTA has limited antimicrobial properties. Several alterations have been made to enhance the biological properties of MTA, such as incorporating nanoparticles made from silver, zinc, gold, and titanium. Therefore, in this study, titanium tetrafluoride (TiF_4_) was added to MTA in an effort to enhance its antimicrobial properties.

Aim

To compare and evaluate the antibacterial efficacy of MTA after the incorporation of TiF_4_.

Materials and methods

A total of 56 samples were made by mixing MTA with different weight proportions of TiF_4_ (1 wt%, 2 wt%, and 3 wt%). Out of these, 28 samples were taken to test each of the following properties: antibacterial efficacy and pH. The specimens were prepared using stainless steel molds of recommended dimensions for testing the pH. The pH was evaluated using a pH meter, and the antibacterial efficacy was assessed using the direct contact test. Data regarding the antibacterial efficacy and pH of MTA with various proportions of TiF_4_ were investigated for normality using the Kolmogorov-Smirnov test and assessed for normal distribution. The antibacterial properties among the four groups were analyzed using one-way analysis of variance (ANOVA), followed by pairwise multiple comparisons using Tukey’s Honest Significant Difference test. The level of statistical significance was determined at p ≤ 0.05. MTA, when incorporated with TiF_4_, showed enhanced antibacterial properties.

Results

On day 1, the group treated with MTA containing 3% TiF_4_ demonstrated the strongest antibacterial effectiveness, with a mean of 4.67 ± 0.04 colony-forming units (CFU)/mL × 10^8. However, the group treated with plain MTA had the lowest mean values, at 5.67 ± 0.25 CFU/mL × 10^8. On day 1, the MTA group with 3% TiF_4_ also had the highest mean pH values (11.90 ± 0.05), while the plain MTA group had the lowest mean pH values (11.64 ± 0.78). On day 7, the MTA group with 3% TiF_4_ had the highest pH value (12.85 ± 0.08), whereas the plain MTA group had the lowest pH value (11.92 ± 0.09).

Conclusion

The inclusion of TiF_4_ resulted in an augmentation of the antibacterial efficacy of MTA against *Enterococcus faecalis* (*E. faecalis*). Hence, the integration of TiF_4_ into MTA can be considered a promising development against *E. faecalis* during endodontic procedures.

## Introduction

Microorganisms play a crucial role in initiating inflammation in the pulp and periapical regions, which is highly significant. Successful endodontic procedures rely on the efficient elimination of these microbes, which includes the use of instruments, thorough irrigation, and intracanal medicament. Various irrigants, intracanal medicaments, and endodontic filling materials contain antimicrobial compounds that help in eliminating these microorganisms. Even after following strict chemo-mechanical procedures, it is possible for bacteria to endure within dentinal tubules, hence presenting a chance for re-infection. The efficacy of endodontic therapy is contingent upon the attainment of an impermeable seal to mitigate the risk of recontamination, as well as the effective reduction or eradication of related microorganisms. Considering that certain biomaterials may not offer an optimal hermetic seal, it is crucial to evaluate their capacity to inhibit bacterial proliferation [1].

The possible antibacterial properties of mineral trioxide aggregate (MTA), which were first proposed in 1993, have been the subject of investigation since 1995. MTA is commonly utilized in several applications, including the repair of root perforations, root-end filling, pulp capping, pulpotomy, and apexification [2]. This material is highly regarded due to its exceptional attributes, including biocompatibility, exceptional sealing ability, and bioactivity. Despite its numerous benefits, MTA has several drawbacks, such as being difficult to handle and necessitating a longer setting time [3]. The MTA material is composed of minute hydrophilic particles that, when exposed to water, undergo a process called colloidal gelation, which builds a solid cement. The two main ions in MTA are phosphorus and calcium. By hydrolyzing calcium silicate components on its surface, calcium hydroxide is liberated, which is responsible for the antibacterial properties of MTA [1].

*Enterococcus faecalis* (*E. faecalis*), a gram-positive, facultative anaerobic coccus, is often encountered in root canals that are linked to recurrent infection and persistent apical periodontitis. This coccus is recognized as an endodontic pathogen. It has been documented that this particular strain of bacteria demonstrates resistance to intracanal medications, such as calcium hydroxide [4].

Prior research on the antibacterial characteristics of MTA has demonstrated positive benefits, although there have been some contradictory findings. Overall, the findings indicate that MTA exhibits restricted antibacterial efficacy. In order to improve the biological characteristics of MTA, many alterations have been implemented, including the integration of nanoparticles derived from silver, zinc, gold, and titanium. Metal fluorides, particularly titanium fluoride, have been increasingly popular in dentistry research because of their distinctive interaction with hard tissue [5].

Titanium tetrafluoride (TiF_4_) has been extensively examined in the field of dentistry for its potential applications: prevention of dental caries, sealing of dentinal tubules in root canals, erosion prevention, mitigation of dentinal hypersensitivity, and as an antibacterial agent. Prior research has demonstrated that the incorporation of TiF_4_ (1 wt% and 2 wt%) into biodentine leads to improvements in its physicochemical characteristics and antibacterial capabilities. There are no studies that include the integration of TiF_4_​ into MTA. Hence, in this investigation, TiF_4_​ was included in MTA with the aim of augmenting its antibacterial characteristics [6]. The null hypothesis posited that the inclusion of TiF_4_​ does not have a substantial impact on the antibacterial capabilities of MTA.

## Materials and methods

Synthesis of TiF_4_

A green and fast synthesis method is used to synthesize TiF_4_. This method involves titanium powder and graphite fluoride as starting materials. Titanium powder and graphite fluoride, with a fluorine stoichiometry (denoted as x) ranging from 0.3 to 1.2, are thoroughly mixed at room temperature according to a ratio of (1 + 0.25x) to 1. This mixture is then compacted into a solid form through cold pressing. The resulting block is subjected to heating under an inert atmosphere, raising the temperature above 560°C. This prompts an exothermic chemical reaction to occur. The gaseous product formed is collected and subsequently cooled, resulting in the formation of TiF_4_​ [7].

A mixture was prepared by combining commercially available MTA powder (Dentsply Tulsa Dental, Tulsa, OK, USA) with different ratios of TiF_4_ powder (Aldrich Chemical Company, Milwaukee, WI, USA). The electronic balance was used to measure three distinct weight proportions of TiF_4_ powder, which were then added to MTA: 1 wt%, 2 wt%, and 3 wt%. The MTA was modified by including TiF_4_ powder and then combined with an amalgamator to achieve a homogeneous mixture. Distilled water was subsequently introduced and blended using a cement spatula to achieve a homogeneous mixture [6].

pH evaluation

A split mold made of stainless steel, with a diameter of 8 ± 0.1 mm and a height of 1.6 ± 0.1 mm, was utilized to properly pack the combined material. The specimens, with a sample size of seven for each group, were promptly submerged in deionized water (10 mL) with a pH of 6.8 in sealed polypropylene containers. The specimens were kept at a temperature of 37°C. The water used for storage was then replenished at designated end-points, namely day 1 and day 7. The pH of the collected water was determined using a pH meter [8].

Antimicrobial efficacy 

Direct contact test (DCT) was utilized to assess the antibacterial efficacy of modified MTA. The test involved uniformly coating the side walls of seven wells in a 96-well microtiter plate with freshly mixed test material, referred to as "plate A" for each group. The surface of each test material was subsequently coated with a 10 µL suspension of *E. faecalis* bacterium, which had a concentration of 3 × 10^8 colony-forming units (CFU)/mL. Plate A was supplemented with Brain Heart Infusion (BHI) broth (235 µL) using a micropipette, establishing an optimal growth environment. After this supplementation, 15 µL of the mixture from each well on plate A was transferred to a separate plate, referred to as "plate B." In plate B, seven adjacent wells were filled with fresh medium (205 µL). The positive control consisted of uncoated wells that were exclusively exposed to bacteria, whereas the negative control consisted of coated wells containing the test materials and BHI broth but without bacteria.

The antibacterial activity was assessed one hour after the samples were mixed and after being cautiously soaked in 280 µL of phosphate-buffered saline for a duration of seven days at a temperature of 37°C. The densitometric measurements were conducted at a depth of 620 nm using a microplate spectrophotometer, set at a temperature of 37°C, to constantly monitor the bacterial expansion in each well. Data were collected at 30-minute intervals over a period of 15 hours [6].

The determination of baseline values involved the subtraction of optical density (OD) readings received from the negative control. The experimental data were documented in OD units, which represented the degree of inhibition or stimulation of bacterial growth relative to the baseline [6]. Densitometric measurements, which offer insights into the magnitude of bacterial proliferation, were recorded on the initial day and on the seventh day for every collection of specimens. This enabled the assessment of bacterial growth patterns over the duration of the experiment [9].

Statistical analysis

Data regarding antibacterial efficacy (CFU/mL × 10^8) and pH for MTA and plain MTA with various proportions of TiF_4_ were entered into Microsoft Excel (Microsoft® Corp., Redmond, WA, USA) and analyzed using IBM SPSS Statistics for Windows, Version 20 (Released 2011; IBM Corp., Armonk, NY, USA). Data were investigated for normality using the Kolmogorov-Smirnov test and assessed for a normal distribution. Descriptive statistics were derived as mean, standard deviation, and 95% confidence interval. The microbiological properties between the four groups were analyzed using one-way analysis of variance (ANOVA), followed by pairwise multiple comparisons using Tukey’s Honest Significant Difference test. The level of statistical significance was determined at p ≤ 0.05.

## Results

The antimicrobial efficacy of the group treated with MTA with 3% TiF_4_ was the highest on day 1, with a mean of 4.67 ± 0.04 CFU/mL × 10^8. In contrast, the group treated with plain MTA exhibited the lowest mean values, with a mean of 5.67 ± 0.25 CFU/mL × 10^8. The MTA with 3% TiF_4_ group exhibited the highest antimicrobial efficacy on day 7, with a concentration of 4.22 ± 0.03 CFU/mL × 10^8. Conversely, the plain MTA group exhibited the lowest efficacy, with a concentration of 5.22 ± 0.18 CFU/mL × 10^8. The mean pH values on day 1 were highest in the MTA group with 3% TiF_4_ (11.90 ± 0.05), while the lowest mean pH values were reported in the plain MTA group (11.64 ± 0.78). The MTA group with 3% TiF_4_ exhibited the highest pH values on day 7, with a value of 12.85 ± 0.08. In contrast, the plain MTA group reported the lowest pH values, with a value of 11.92 ± 0.09. As time progressed, the pH measurements demonstrated a progressive upward trend (Tables 1-2).

**Table 1:** Intergroup comparison of the microbiological properties of the materials * Statistically significant (p < 0.05) Group I: MTA; Group II: MTA + 1% TiF_4_; Group III: MTA + 2% TiF_4_; Group IV: MTA + 3% TiF_4_; F-value: One way ANOVA test value MTA: Mineral trioxide aggregate; TiF_4_: Titanium tetrafluoride; ANOVA: Analysis of variance; CFU: Colony-forming units

Physical properties			Mean + SD	95% confidence interval for mean		F-value (p-value)
				Lower	Upper	
Antibacterial efficacy (CFU/mL × 10^8)	Day 1 (n = 28)	Group I	5.67 + 0.25	5.44	5.91	78.237 (0.000)*
		Group II	4.92 + 0.08	4.84	4.99	
		Group III	4.78 + 0.05	4.73	4.83	
		Group IV	4.67 + 0.04	4.63	4.71	
		Total	5.01 + 0.42	4.85	5.17	
	Day 7 (n = 28)	Group I	5.22 + 0.18	5.05	5.39	137.370 (0.000)*
		Group II	4.40 + 0.08	4.32	4.48	
		Group III	4.30 + 0.01	4.28	4.31	
		Group IV	4.22 + 0.03	4.19	4.25	
		Total	4.53 + 0.41	4.37	4.70	
pH	Day 1 (n = 28)	Group I	10.36 + 0.23	10.14	10.57	263.037 (0.000)*
		Group II	9.65 + 0.23	9.43	9.87	
		Group III	8.26 + 0.21	8.06	8.46	
		Group IV	7.23 + 0.23	7.01	7.44	
		Total	8.87 + 1.25	8.39	9.36	
	Day 7 (n = 28)	Group I	9.94 + 0.17	9.78	10.10	471.604 (0.000)*
		Group II	9.63 + 0.13	9.50	9.75	
		Group III	7.85 + 0.07	7.78	7.92	
		Group IV	7.44 + 0.19	7.26	7.62	
		Total	8.72 + 1.11	8.28	9.15	

**Table 2:** Post hoc Tukey’s test for intergroup comparison of the microbiological properties of the materials * Mean difference is significant at the 0.05 level Group I: MTA; Group II: MTA + 1% TiF_4_; Group III: MTA + 2% TiF_4_; Group IV: MTA + 3% TiF_4_ MTA: Mineral trioxide aggregate; TiF_4_: Titanium tetrafluoride; CFU: Colony-forming units

Dependent variable	Groups (I)	Groups (J)	Mean difference (I-J)	p-value	95% confidence interval	
					Lower	Upper
Antibacterial efficacy at day 1 (CFU/mL × 10^8)	Group I	Group II	0.75714*	0.000	0.5566	0.9577
		Group III	0.89429*	0.000	0.6937	1.0949
		Group IV	1.00714*	0.000	0.8066	1.2077
	Group II	Group III	0.13714	0.260	-0.0634	0.3377
		Group IV	0.25000*	0.011	0.0494	0.4506
	Group III	Group IV	0.11286	0.424	-0.0877	0.3134
Antibacterial efficacy at day 7 (CFU/mL × 10^8)	Group I	Group II	0.81714*	0.000	0.6635	0.9708
		Group III	0.92143*	0.000	0.7678	1.0751
		Group IV	0.99571*	0.000	0.8421	1.1493
	Group II	Group III	0.10429	0.266	-0.0493	0.2579
		Group IV	0.17857*	0.018	0.0249	0.3322
	Group III	Group IV	0.07429	0.551	-0.0793	0.2279
pH at day 1	Group I	Group II	0.70571*	0.000	0.3686	1.0428
		Group III	2.09286*	0.000	1.7557	2.4300
		Group IV	3.13143*	0.000	2.7943	3.4686
	Group II	Group III	1.38714*	0.000	1.0500	1.7243
		Group IV	2.42571^*^	0.000	2.0886	2.7628
	Group III	Group IV	1.03857*	0.000	0.7014	1.3757
pH at day 7	Group I	Group II	0.31571*	0.004	0.0907	0.5407
		Group III	2.09143*	0.000	1.8664	2.3165
		Group IV	2.50143*	0.000	2.2764	2.7265
	Group II	Group III	1.77571*	0.000	1.5507	2.0007
		Group IV	2.18571*	0.000	1.9607	2.4107
	Group III	Group IV	0.41000*	0.000	0.1850	0.6350

## Discussion

The dental pulp and periodontium are connected via the lateral canal and apical foramen, respectively. Pulpal and periradicular pathosis develop when germs are exposed to the dental pulp and periradicular tissues. Researchers using experimental animals have demonstrated the formation of lesions when these tissues were exposed to microbes, and the absence of pulpal and periradicular pathosis in microbe-free environments [10].

In order to achieve successful endodontic therapy, it is crucial to completely eliminate microorganisms from the pulp space. Insufficient cleaning and shaping of the root canal system, as well as inadequate obturation, frequently lead to failures in post-endodontic situations. Microorganisms, particularly *E. faecalis*, have been detected in 35% to 100% of unsuccessful cases [9].

It is widely recognized that *E. faecalis* is resistant to endodontic infection. While *E. faecalis* may comprise only a minute fraction of the root canal flora during initial endodontic infections, it is the most frequently detected microorganism in endodontically treated teeth [11].

Moreover, the persistence of microorganisms organized as microbial biofilm is associated with endodontic treatment failure; therefore, it is critical to assess the antimicrobial activity of the materials against this bacterial organization [12]. Consequently, the antimicrobial characteristics of the materials and cements utilized in root canal therapy are crucial. Because *E. faecalis* is a gram-positive bacterium frequently encountered in drug-resistant apical periodontitis, and its eradication is often difficult, it has been the reference microorganism in all dental research concentrating on antimicrobial agents [13]. The approach outlined by Guerreiro-Tanomaru et al. permits the direct examination of materials with biofilm-forming *E. faecalis* strains [14].

MTA is a dental material that finds extensive application in the treatment of root perforation, retrograde fillings, and exposed apices in teeth. MTA is biocompatible, has the right physicochemical characteristics, and can promote bone mineralization [15]. Tri- and di-calcium silicates are the primary constituents of MTA. An alkaline calcium silicate gel is produced by the hydration of these components. Within a silicate matrix, hydroxide ions are released from calcium hydroxide. An environment that is highly alkaline, due to the presence of hydroxide ions, is detrimental to microbial growth. Nevertheless, there exists a contentious debate regarding its antibacterial characteristics [16].

The significance of assessing the antibacterial efficacy of materials against *E. faecalis* in cases of endodontic treatment failure is underscored by its high prevalence. The inclusion of silver-containing zeolite was investigated in a previous study conducted by Luddin and Ahmed. The addition of chlorhexidine to materials such as glass ionomer cement (GIC), resins, and MTA has been found to enhance their antibacterial properties [17]. Certain studies substituted alternative liquids for distilled water when combining MTA powder with the intention of augmenting its antimicrobial properties. The findings suggest that augmenting the antibacterial capability of MTA through the addition of different substances could potentially have detrimental effects on the material's other characteristics [18]. To date, a range of metallic nanoparticles, including silver, gold, zinc, titanium, and bismuth, have exhibited antibacterial characteristics [19]. A study by Lim and Yoo concluded that adding CaF_2_ significantly enhances antibacterial activity against *E. faecalis*, *Porphyromonas endodontalis* (*P. endodontalis*), and *Porphyromonas gingivalis* (*P. gingivalis*), compared to pure MTA, both in its powder form and its eluate [20]. As titanium is a nontoxic element, TiF_4_ has also not been associated with any adverse effects. Titanium ion concurrently binds to organic material on the enamel surface, as well as fluoride and apatite in enamel and dentin, exhibiting a remarkable complex-binding capacity [21].

According to Weiss et al., the DCT is a quantitative and reproducible technique meant to mimic the interaction between microorganisms and materials in the root canal. When carryover controls are used, this method provides more control over confounding circumstances than the agar diffusion test and enables the assessment of the materials' bactericidal effect [19]. However, one disadvantage of the DCT is that it exposes the microtiter plate to a variety of conditions during the experimental setup, which raises the possibility of contamination [22].

In the present investigation, different ratios of TiF_4_ were included in MTA to augment its antibacterial efficacy. The samples were divided into four groups based on the various proportions of TiF_4_ (1 wt%, 2 wt%, and 3 wt%) incorporated. The group treated with MTA with 3% TiF_4_ had the highest mean values of antimicrobial efficacy on day 1, with a mean of 4.67 ± 0.04 CFU/mL × 10^8. Conversely, the group treated with plain MTA had the lowest mean values, with a mean of 5.67 ± 0.25 CFU/mL × 10^8. The antimicrobial efficacy on day 7 was highest in the MTA with 3% TiF_4_ group, with a concentration of 4.22 ± 0.03 CFU/mL × 10^8, whereas the lowest efficacy was seen in the plain MTA group, with a concentration of 5.22 ± 0.18 CFU/mL × 10^8. The findings indicated that the incorporation of 3% TiF_4_ into MTA demonstrated the most pronounced antibacterial effectiveness on both day 1 and day 7, with notable variations observed among the different groups.

The findings derived from our investigation indicate that the antimicrobial efficacy of MTA is enhanced when the concentration of TiF_4_ increases. The antibacterial activity of MTA, when combined with different ratios of TiF_4_, can be attributed to the process of ionization, which results in the release of hydroxyl ions and subsequently leads to an elevation in pH levels. The pH can either irreversibly or reversibly deactivate the cellular membrane enzymes of the microorganism [4].

Also, the pH level of MTA plays a significant role in its interaction with neighboring tissues. In our investigation, it was observed that all the groups exhibited an alkaline pH, which aligns with the results reported in prior research. An alkaline pH is commonly observed as a result of the liberation of hydroxyl ions during the process of hydration. The induction of hard tissue formation can be facilitated by an alkaline pH, while the influence of hydroxyl ions on bacterial cell membranes can impact cell metabolism, growth, and division. On day 1 and day 7, the pH values were measured, revealing differences across varied weight proportions of TiF_4_ [8].

The highest mean pH values on day 1 were seen in the MTA group with 3% TiF_4_ (11.90 ± 0.05), whereas the lowest mean pH values were reported in the plain MTA group (11.64 ± 0.78). The highest pH values on day 7 were seen in the MTA group with 3% TiF_4_, with a value of 12.85 ± 0.08. Conversely, the lowest pH values were reported in the plain MTA group, with a value of 11.92 ± 0.09. The pH measurements exhibited a progressive upward trend as time progressed. The addition of TiF_4_ to MTA resulted in a greater concentration of hydroxyl ions in the medium, leading to an elevation in pH. While the various pH values observed in this study deviated from those reported in other research, this disparity may be attributed to difficult-to-manage elements and the absence of standardized techniques for quantifying MTA characteristics [8].

A limitation of this study is that the DCT used for testing antibacterial efficacy does not permit the assessment of bacteria under biofilm conditions. The current work standardized the collection of microorganisms to the mid-exponential growth phase. Subsequent research could employ models incorporating bacteria at various stages of growth, as it has been demonstrated that *E. faecalis* strains exhibit greater vulnerability to endodontic medicaments during the exponential growth phase, as opposed to the stationary and starving phases. 

In summary, MTA with TiF_4_ incorporation showed antibacterial and antibiofilm properties. After endodontic treatment, this novel substance is even capable of combating reinfections. While our findings are encouraging, further research is required to establish the efficacy and safety profile of MTA combined with TiF_4_ in endodontic patients over an extended period of time [5].

## Conclusions

Based on the data obtained from this study, it can be inferred that the inclusion of TiF_4_ resulted in an augmentation of the antibacterial efficacy of MTA against *E. faecalis*. An increase in the proportions of TiF_4_ added resulted in an increase in antibacterial efficacy. Hence, the integration of TiF_4_ into MTA can be considered a promising approach against *E. faecalis* during endodontic procedures.

## References

[1] Asgary S, Akbari Kamrani F, Taheri S (2007). Evaluation of antimicrobial effect of MTA, calcium hydroxide, and CEM cement. Iran Endod J.

[2] Chaudhari PS, Chandak MG, Jaiswal AA, Mankar NP, Paul P (2022). A breakthrough in the era of calcium silicate-based cements: a critical review. Cureus.

[3] Ravindran V, Jeevanandan G (2023). Comparative evaluation of the physical and antimicrobial properties of mineral trioxide aggregate, biodentine, and a modified fast-setting mineral trioxide aggregate without tricalcium aluminate: an in vitro study. Cureus.

[4] Koruyucu M, Topcuoglu N, Tuna EB, Ozel S, Gencay K, Kulekci G, Seymen F (2015). An assessment of antibacterial activity of three pulp capping materials on Enterococcus faecalis by a direct contact test: an in vitro study. Eur J Dent.

[5] Hernandez-Delgadillo R, Del Angel-Mosqueda C, Solís-Soto JM (2017). Antimicrobial and antibiofilm activities of MTA supplemented with bismuth lipophilic nanoparticles. Dent Mater J.

[6] Elsaka SE, Elnaghy AM, Mandorah A, Elshazli AH (2019). Effect of titanium tetrafluoride addition on the physicochemical and antibacterial properties of Biodentine as intraorfice barrier. Dent Mater.

[7] (2014). Titanium tetrafluoride green and fast synthesis method. https://patents.google.com/patent/CN103539200B/en.

[8] Bernardi A, Bortoluzzi EA, Felippe WT, Felippe MC, Wan WS, Teixeira CS (2017). Effects of the addition of nanoparticulate calcium carbonate on setting time, dimensional change, compressive strength, solubility and pH of MTA. Int Endod J.

[9] Ghatole K, Patil A, Giriyappa RH, Singh TV, Jyotsna SV, Rairam S (2016). Evaluation of antibacterial efficacy of MTA with and without additives like silver zeolite and chlorhexidine. J Clin Diagn Res.

[10] Torabinejad M, Chivian N (1999). Clinical applications of mineral trioxide aggregate. J Endod.

[11] Al-Hezaimi K, Al-Shalan TA, Naghshbandi J, Oglesby S, Simon JH, Rotstein I (2006). Antibacterial effect of two mineral trioxide aggregate (MTA) preparations against Enterococcus faecalis and Streptococcus sanguis in vitro. J Endod.

[12] Vazquez-Garcia F, Tanomaru-Filho M, Chávez-Andrade GM, Bosso-Martelo R, Basso-Bernardi MI, Guerreiro-Tanomaru JM (2016). Effect of silver nanoparticles on physicochemical and antibacterial properties of calcium silicate cements. Braz Dent J.

[13] Jonaidi-Jafari N, Izadi M, Javidi P (2016). The effects of silver nanoparticles on antimicrobial activity of ProRoot mineral trioxide aggregate (MTA) and calcium enriched mixture (CEM). J Clin Exp Dent.

[14] Guerreiro-Tanomaru JM, de Faria-Júnior NB, Duarte MA, Ordinola-Zapata R, Graeff MS, Tanomaru-Filho M (2013). Comparative analysis of Enterococcus faecalis biofilm formation on different substrates. J Endod.

[15] Guerreiro Tanomaru JM, Storto I, Da Silva GF, Bosso R, Costa BC, Bernardi MI, Tanomaru-Filho M (2014). Radiopacity, pH and antimicrobial activity of Portland cement associated with micro- and nanoparticles of zirconium oxide and niobium oxide. Dent Mater J.

[16] Khedmat S, Aminipor M, Pourhajibagher M, Kharazifar MJ, Bahador A (2018). Comparison of antibacterial activities of ProRoot MTA, OrthoMTA, and RetroMTA against three anaerobic endodontic bacteria. J Dent (Tehran).

[17] Luddin N, Ahmed HM (2013). The antibacterial activity of sodium hypochlorite and chlorhexidine against Enterococcus faecalis: a review on agar diffusion and direct contact methods. J Conserv Dent.

[18] Samiei M, Aghazadeh M, Lotfi M, Shakoei S, Aghazadeh Z, Vahid Pakdel SM (2013). Antimicrobial efficacy of mineral trioxide aggregate with and without silver nanoparticles. Iran Endod J.

[19] Sen BH, Büyükyilmaz T (1998). The effect of 4% titanium tetrafluoride solution on root canal walls-a preliminary investigation. J Endod.

[20] Lim M, Yoo S (2022). The antibacterial activity of mineral trioxide aggregate containing calcium fluoride. J Dent Sci.

[21] Lovato KF, Sedgley CM (2011). Antibacterial activity of endosequence root repair material and proroot MTA against clinical isolates of Enterococcus faecalis. J Endod.

[22] Weiss EI, Shalhav M, Fuss Z (1996). Assessment of antibacterial activity of endodontic sealers by a direct contact test. Endod Dent Traumatol.

